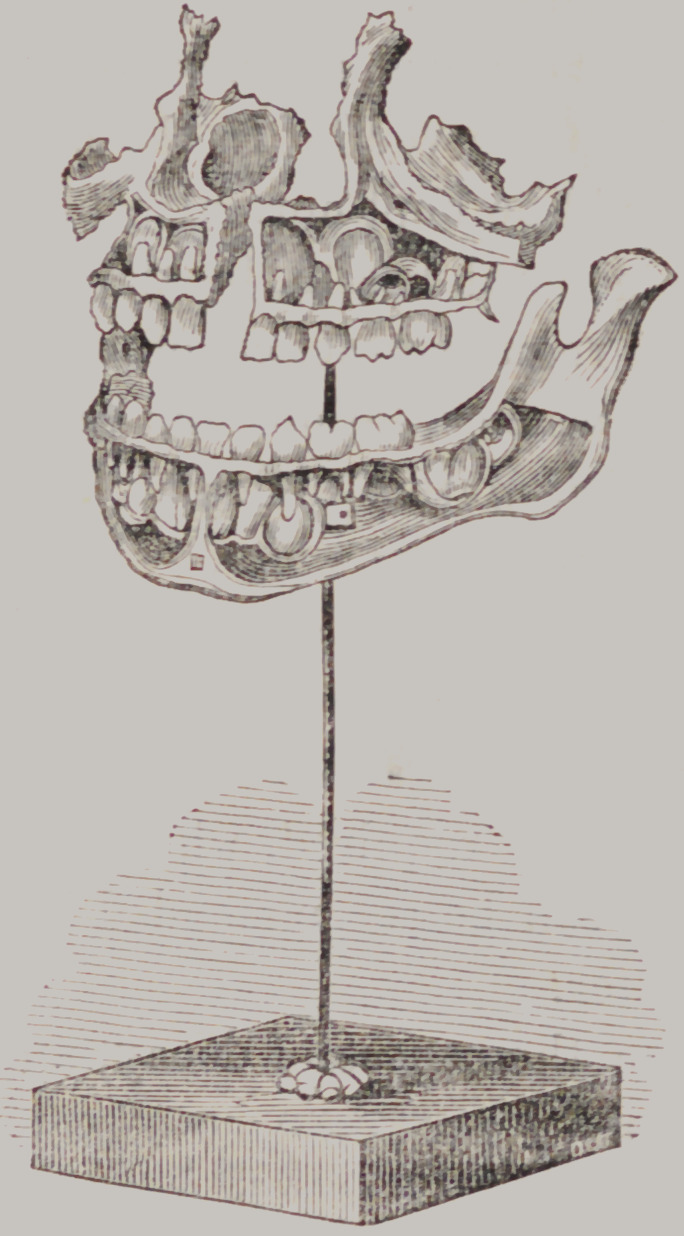# Physiological Observations on the Teeth

**Published:** 1858-11

**Authors:** T. E. St. John


### THE DENTAL REPORTER.

Vol. 1 NOVEMBER, 1858. No. 3.

### PHYSIOLOGICAL OBSERVATIONS ON THE TEETH.

BY PROF. T. E. ST. JOHN.

It is well understood by all who have given any attention to the subject, that in order to insure a healthy denture in the child, it is necessary that the mother be supplied during gestation with a good proportion of those more important materials which are found to enter so largely into the formation of bone and tooth structure. This was briefly noticed in the last number of the *Reporter*, and in continuation of that subject I shall present some considerations as to the best means of *cultivating* and preserving a healthy denture.

It is but a few months of the earlier existence of the child that it is entirely dependent upon the mother for its food, and usually after the eruption of the incisor teeth it maintains a comparatively independent existence, and hence it is necessary that a good degree of care be taken to provide it with that kind and quality of food which may contain the requisite proportion of all the substances required in the formation of a strong frame and vigorous constitution. There are, no doubt, many cases in which disease is hereditary, and I have no hesitency in asserting that in many instances a disease, or liability to disease, is

*enforced* upon the child by an improper connection, or an incompatibility of temperament on the part of the pa: ents, even though the parent may be entirely free from any such disease; but it is a fact that is evident to all, that a large amount of suffering and constitutional derangements and defects are caused by improper food, and a want of any degree of care or attention during the first years of life.

It is at this period that a large proportion of the earthy constituents are provided, for the completion of the skeleton, the extremities are lengthened and become stronger and more solid, and the substance of the teeth is deposited in the gelatinous cells, hence the necessity of exercising great care in the selection of proper nourishment. The child at this time must have more nourishment than that which it can obtain from the breast, and the presence of the teeth is an indication that more solid food is required to supply the nutritive powers during the increased action of growth that is now going on. It is one of the laws of our being, that no organ or structure is furnished until there is use for it in the animal economy, and then it is necessary for the preservation of that organ that it be exercised.

There are many who suppose that the processes of growth in the infant is something over which they have no control, or with which they have nothing to do, but that nature will provide all the wants of the animal economy and complete the various parts of this wonderful organization, regardless of any assisting efforts which may be made. This is an error; nature does nothing but deface, unless provided with, or aided by the necessary materials for reproduction. The machinist can not construct the most simple machine without a supply of tools and material with which to work, and neither can nature form bones or teeth without earthy matter. The period from the first existence of the child until puberty is marked by a continual deposition of osseous structure, and whether that shall be of a perfect and healthy character depends entirely upon the nutritive material which is supplied to the individual.

The permanent teeth are formed during the brief existence of the temporary set, and if the latter are perfect in their organization there will usually be no difficulty in the subsequent eruption, and regular arrangement of the permanent set. The relation that exists between the two sets is very beautifully shown in the adjoining original cut, which was taken from a specimen in the possession of Mr. J. T. Toland of this city.

In order to secure a healthy permanent denture, it is necessary that the temporary teeth be preserved as long as possible, and should they be attacked with caries it is advised by the best dental authority to have them filled. Great care, however, should be exercised in filling the deciduous teeth, as they are less capable of sustaining pressure than the permanent set. Dr. Taft at the fourth annual meeting of the American Dental Convention, recommended filling the temporary teeth when the decay occurs three or four years previous to the period of shedding, as the attachments are then sufficiently strong to resist the pressure accompanying the operation, which, he says, need not be greater than that in mastication. He remarked that great care should be taken to prevent the destruction of the deciduous pulp, as the parts around the tooth are less likely to remain healthy after the death of the pulp, particularly of the temporary teeth.

It will be noticed in some instances that the deciduous teeth are not thrown off at the proper time, and that they present a barrier to the eruption of the succeeding one, causing it to be thrown from its natural course and present more or less irregularity; in such a case the best and only course is to remove the temporary tooth. Care should be taken during the eruption of the permanent teeth to prevent irregularities, as this is one of the most fruitful causes of premature decay. It is necessary that all pressure of the teeth upon each other be prevented as much as may be, so that each shall occupy its respective place, independent of its neighbor, and if slight spaces exist between them, they may be preserved much better, as in that case the lodgement of all corosive matter will be effectually prevented by a judicious use of the brush after each meal.

Having procured a healthy permanent denture, the next question of importance is, how can they be preserved? In the first place, the secretions of the mouth should be kept normal, which can only be done by attention to the general health, by the use of proper and wholesome food, and a regular practice of exercise and bathing. The use of mouth washes, lotions and tooth powders are entirely useless in *correcting* this difficulty, and when they are used, the best application is *pure water*.

It must also be borne in mind *that entire and absolute cleanliness of the mouth is indispensably necessary, if we would have healthy dentures.* To obtain this a thorough and frequent application of the brush is the only means required, while as a tooth powder the most beneficial that can be recommended is a small quantity of castile soap and water.

In the next number I shall notice the causes of decay, and the best means to prevent its ravages.

## Figures and Tables

**Figure f1:**